# Improved reverse Monte Carlo analysis of optical property of Fe and Ni from reflection electron energy loss spectroscopy spectra

**DOI:** 10.1038/s41598-023-38769-4

**Published:** 2023-08-01

**Authors:** Z. Li, J. M. Gong, B. Da, J. Tóth, K. Tőkési, R. G. Zeng, Z. J. Ding

**Affiliations:** 1 Department of Physics, University of Science and Technology of China, Hefei, 230026 Anhui People’s Republic of China; 2 Center for Basic Research on Materials, National Institute for Materials Science, Tsukuba, Ibaraki 305-0044 Japan; 3 Institute for Nuclear Research, P.O. Box 51, Debrecen, Hungary; 4 Institute of Materials, China Academy of Engineering Physics, P.O. Box 9071, Jiangyou, 621907 Sichuan People’s Republic of China; 5 Hefei National Laboratory for Physical Science at Microscale, University of Science and Technology of China, Hefei, 230026 Anhui People’s Republic of China

**Keywords:** Electronic properties and materials, Computational methods

## Abstract

The energy loss functions (ELFs) of Fe and Ni have been derived from measured reflection electron energy loss spectroscopy (REELS) spectra by a reverse Monte Carlo analysis in our previous work. In this work, we present further improvements of ELFs for these metals. For Fe, we have updated ELFs at primary electron energies of 2 keV and 3 keV in a wider photon energy region (0–180 eV) with a better accuracy, which is verified by sum rules. Regarding to Ni, we supplement the ELF at primary energy of 5 keV and we also improve the data accuracy at 3 keV. Applying these new and more accurate ELFs we present the optical constants and dielectric functions for the two metals. The improvements were highlighted by comparing our present results with the previous data.

## Introduction

Iron and nickel are the main elements of the earth’s core. The study of the optical properties of these two transition metals has a long period of history. Most optical data of solid substances and compounds included in Palik’s database^1,2^ are obtained by optical methods with a light beam, such as, reflection spectroscopy, absorption spectroscopy and spectroscopic ellipsometry. Another technique differing from the optical methods is to use electron energy loss spectroscopy (EELS)^3,4^ in a transmission electron microscope. However, such a technique has some limitations for its strict experimental conditions including extremely high incident beam energy and a very thin and free-standing sample. These extreme conditions had resulted in that the attention is turned to the reflection mode of EELS, i.e. the reflection electron energy loss spectroscopy (REELS), with a surface electron spectrometer. Without the special requirements for the thickness of samples, the REELS experiments can be performed for a bulk solid. In addition, the incident electron beam energy is just about several hundreds or thousands of eV.

In order to analyze experimental REELS spectra, many theoretical approaches have been proposed in the past years^5–15^ which emphasized the theoretical description of the inelastic scattering in the surface region by including both the bulk and surface contributions. The analytical models for the background removal in REELS spectrum analysis have some shortcomings although they are helpful to understand the signal formation mechanism during the interaction between electrons and solid. On the one hand, there will be logical contradiction between input parameters and calculation results. Because the electron inelastic mean free path (IMFP) and the surface excitation parameter should be known in advance as input parameters, but in fact these parameters need to be determined by the ELF of the materials. What is more, the surface excitation of the sample is actually related to the depth, rather than the uniform scattering assumed by the algorithm^15^. In addition, the ratio between the elastic and the inelastic scattering cross sections has a great influence on the derived ELF and the shape of the simulated REELS spectrum.

To solve these problems, Da et al. developed a reverse Monte Carlo (RMC) technique for deriving the effective ELF from measured REELS spectra at different electron energies^16,17^. They successfully determined ELF, optical constants and dielectric function of SiO_2_, where the extracted data were verified with the oscillator-strength (*f*-) and perfect-screening (*ps*-) sum rules^18^. The RMC combines a well-established Monte Carlo simulation method for the electron interaction with solids and a Markov chain Monte Carlo (MCMC) iterative updating of a parameterized ELF. In the iterative process the parameterized ELF was employed to calculate the required differential inverse IMFP (DIIMFP) for a Monte Carlo simulation of REELS spectrum. The simulated spectrum was then compared with experimentally measured spectrum for further optimization of ELF. Xu et al.^19–21^ later have improved the RMC method by employing a more exact physical model of electron inelastic scattering in the surface region. They considered a depth-dependent DIIMFP including both the bulk excitation and the inhomogeneous surface excitation for electron inelastic scattering in vacuum and inside the sample surface region by a semi-classical approach^22^. This method has been successfully applied to obtain the absolute ELFs for some other solids^23–28^ with our CTMC-RMC code^29^.

Xu et al. have studied the optical properties of Fe^19^ and Ni^20^ by employing the RMC method. Their sum rules results were obviously improved when compared with previous results. In this work, we further improve the values of ELFs by the RMC method, and then improve the optical constants and dielectric function according to the new ELFs. For Fe, we have considered a wider energy loss range (0–180 eV) in our simulation of REELS rather than the 0–100 eV range in Xu’s work^19^. We show that our present ELFs are more accurate at the primary electron energies of 2 and 3 keV than the previous ones. Regarding to Ni, we improve the accuracy of the ELF at 3 keV primary energy, in which the goodness of *f*-sum rule of ELF increased by nearly 50%. Moreover, we supplement the values of ELF at higher primary energy of 5 keV, for which even better sum rule values were obtained than 3 keV primary energy. In addition, our present dielectric function of Ni in infrared region (below 1.6 eV) is more reasonable than Xu et al.^20^ for their real part of dielectric function conflicts with the prediction of Drude dielectric function model in the low frequency region.

## Experiment

Optical properties are inherent bulk properties independent of primary electron energy. However, we usually perform more than one measurement for the investigated material to verify the obtained ELF. In this work, the REELS spectra of Fe sample were recorded at primary electron energies of 2 and 3 keV, and those of the Ni sample were recorded at primary electron energies of 3 and 5 keV by a home-built electron spectrometer ESA-31 developed in ATOMKI^30^. During the experiments we used the fixed retardation ratio mode with a relative energy resolution of 5 × 10^–3^. The angle of incident primary electron beam and analyzed electrons are 50° and 0° with respect to the surface normal, respectively. The other experimental details have been described previously^19,20^. The same experimental conditions and process were performed except that the recorded REELS spectra in this work were presented in the energy loss range of 0–180 eV for both Fe and Ni rather than 0–100 eV for Fe^19^ and 0–200 eV for Ni^20^.

## Theory

### Reverse Monte Carlo method

The Monte Carlo modeling of electron-solid interactions for a REELS spectrum is the basis of the RMC method, which uses Mott’s cross section^31^ for electron elastic scattering and the dielectric function theory for inelastic scattering.

The relativistic expression of the differential elastic cross section, i.e., the Mott’s cross section, is expressed by the following formula,

1 $$\frac{{{\text{d}}\sigma }}{{{\text{d}}\Omega }} = \left| {f\left( \theta \right)} \right|^{2} + \left| {g\left( \theta \right)} \right|^{2} ,$$

where $\theta$ is the scattering angle, and the scattering amplitudes calculated by the partial wave expansion method^24^ can be described as:

2 $$\begin{aligned} f\left( \theta \right) & = \frac{1}{{2iK}}\sum\limits_{l} {\left\{ {\left( {l + 1} \right)\left[ {\exp \left( {2i\delta _{l}^{ + } } \right) - 1} \right] + l\left[ {\exp \left( {2i\delta _{l}^{ - } } \right) - 1} \right]} \right\}} P_{l} \left( {\cos \theta } \right); \\ g\left( \theta \right) & = \frac{1}{{2iK}}\sum\limits_{l} {\left[ {\exp \left( {2i\delta _{l}^{ - } } \right) - \exp \left( {2i\delta _{l}^{ + } } \right)} \right]} P_{l}^{1} \left( {\cos \theta } \right), \\ \end{aligned}$$

where $K$ is the relativistic wave vector of electron, $P_{l} \left( {\cos \theta } \right)$ and $P_{l}^{1} \left( {\cos \theta } \right)$ are the Legendre and the first-order associated Legendre functions, $\delta_{l}^{ + }$ and $\delta_{l}^{ - }$ are spin-up and spin-down phase shifts of the $l$th partial wave, respectively. The Thomas–Fermi–Dirac atomic potential^32^ is employed in the numerical calculation of the phase shifts.

The dielectric function formalism is the most commonly used theoretical method to describe the electron inelastic scattering process. Owing to the surface boundary at the interface between bulk material and vacuum, in the process of simulating REELS by Monte Carlo method, not only the bulk excitation mode but also the surface excitation mode should be considered. A quantum mechanical calculation of electron inelastic scattering cross section near the surface region^9,10^ has enabled the successful theoretical simulation of REELS spectrum for a metal with the known optical ELF^11,12^. However, considering the computation cost a semi-classical model is applied in this work for the electron inelastic scattering process. The depth-dependent DIIMFP, which can fully describe the surface excitation for an electron penetrating the surface from solid/vacuum side into the vacuum/solid side, is derived as^22^:

3 $$\begin{aligned} \sigma(z) = & \frac{2}{{\pi v^{2} }}\mathop \smallint \nolimits_{{q_{ - } }}^{{q_{ + } }} {\text{d}}q\frac{1}{q}{\text{Im}} \left[ {\frac{ - 1}{{\varepsilon \left( {{\mathbf{q}},\omega } \right)}}} \right]\Theta \left( { - z} \right) \\ &+ \frac{4\cos \alpha }{{\pi^{3} }}\mathop \smallint \nolimits_{{q_{ - } }}^{{q_{ + } }} {\text{d}}q\mathop \smallint \nolimits_{0}^{{\frac{\pi }{2}}} {\text{d}}\theta \mathop \smallint \nolimits_{0}^{2\pi } {\text{d}}\phi \frac{{q\sin^{2} \theta \cos \left( {q_{ \bot } z} \right)\exp \left( {q_{\parallel } z} \right)}}{{\tilde{\omega }^{2} + q_{\parallel }^{2} v_{ \bot }^{2} }} \\ & \times \left\{ {{\text{Im}} \left[ {\frac{ - 1}{{\varepsilon \left( {{\mathbf{q}}_{\parallel } ,\omega } \right) + 1}}} \right] - \frac{1}{2}{\text{Im}} \left[ {\frac{ - 1}{{\varepsilon \left( {{\mathbf{q}}_{\parallel } ,\omega } \right)}}} \right]} \right\}\Theta \left( { - z} \right)\quad v_{ \bot } > 0, \\ & + \frac{4\cos \alpha }{{\pi^{3} }}\mathop \smallint \nolimits_{{q_{ - } }}^{{q_{ + } }} {\text{d}}q\mathop \smallint \nolimits_{0}^{{\frac{\pi }{2}}} {\text{d}}\theta \mathop \smallint \nolimits_{0}^{2\pi } {\text{d}}\phi \frac{{q\sin^{2} \theta \exp \left( { - q_{\parallel } z} \right)}}{{\tilde{\omega }^{2} + q_{\parallel }^{2} v_{ \bot }^{2} }} \\ & \times {\text{Im}} \left[ {\frac{ - 1}{{\varepsilon \left( {{\mathbf{q}}_{\parallel } ,\omega } \right) + 1}}} \right]\left[ {2\cos \left( {\frac{{\tilde{\omega }z}}{v\cos \alpha }} \right) - \exp \left( { - q_{\parallel } z} \right)} \right]\Theta \left( z \right) \\ \end{aligned}$$

and

4 $$\begin{aligned} \sigma (z) = & \frac{2}{{\pi v^{2} }}\mathop \smallint \nolimits_{{q_{ - } }}^{{q_{ + } }} {\text{d}}q\frac{1}{q}{\text{Im}} \left[ {\frac{ - 1}{{\varepsilon \left( {{\mathbf{q}},\omega } \right)}}} \right]\Theta \left( { - z} \right) \\ & + \frac{4\cos \alpha }{{\pi^{3} }}\mathop \smallint \nolimits_{{q_{ - } }}^{{q_{ + } }} {\text{d}}q\mathop \smallint \nolimits_{0}^{{\frac{\pi }{2}}} {\text{d}}\theta \mathop \smallint \nolimits_{0}^{2\pi } {\text{d}}\phi \frac{{q\sin^{2} \theta \cos \left( { - q_{ \bot } z} \right)\exp \left( { - q_{\parallel } z} \right)}}{{\tilde{\omega }^{2} + q_{\parallel }^{2} v_{ \bot }^{2} }} \\ & \times {\text{Im}} \left[ {\frac{ - 1}{{\varepsilon \left( {{\mathbf{q}}_{\parallel } ,\omega } \right) + 1}}} \right]\Theta \left( z \right) + \frac{4\cos \alpha }{{\pi^{3} }}\mathop \smallint \nolimits_{{q_{ - } }}^{{q_{ + } }} {\text{d}}q\mathop \smallint \nolimits_{0}^{{\frac{\pi }{2}}} {\text{d}}\theta \mathop \smallint \nolimits_{0}^{2\pi } {\text{d}}\phi \, \quad v_{ \bot } < 0. \\ & \times \frac{{q\sin^{2} \theta \exp \left( {q_{\parallel } z} \right)}}{{\tilde{\omega }^{2} + q_{\parallel }^{2} v_{ \bot }^{2} }}\left\{ {{\text{Im}} \left[ {\frac{ - 1}{{\varepsilon \left( {{\mathbf{q}}_{\parallel } ,\omega } \right) + 1}}} \right] - \frac{1}{2}{\text{Im}} \left[ {\frac{ - 1}{{\varepsilon \left( {{\mathbf{q}}_{\parallel } ,\omega } \right)}}} \right]} \right\} \\ & \times \left[ {2\cos \left( {\frac{{\tilde{\omega }z}}{v\cos \alpha }} \right) - \exp \left( {q_{\parallel } z} \right)} \right]\Theta \left( { - z} \right) \\ \end{aligned}$$

The atomic unit is used in the above discussion, that is to say, the electron rest mass, electron charge and reduced Planck constant are all set to 1 ($m_{e} = e = \hbar = 1$). In Eqs. (3) and (4), $E = {{v^{2} } \mathord{\left/ {\vphantom {{v^{2} } 2}} \right. \kern-0pt} 2}$, $\tilde{\omega } = \omega - qv{\kern 1pt} {\kern 1pt} {\text{sin}}\theta {\kern 1pt} {\kern 1pt} {\text{cos}}\phi {\kern 1pt} {\kern 1pt} {\text{sin}}\alpha$, $q_{||} = q\sin \theta$, $v_{ \bot } = v\cos \alpha$, where $\alpha$ is the angle between incident electron and surface normal, $\phi$ is spherical coordinate integral variable. $\Theta ( - z)$ and $\Theta (z)$ in the end of each item are step functions about depth $z$. The upper and lower limits of $q$ in the integrals are $q_{ \pm } = \sqrt {2E} \pm \sqrt {2(E - \omega )}$. Equations (3) and (4) of the DIIMFP containing both the bulk- and surface-ELF terms (i.e. ${\text{Im}}\left\{ {{{ - 1} \mathord{\left/ {\vphantom {{ - 1} {\varepsilon \left( {{\mathbf{q}},\omega } \right)}}} \right. \kern-0pt} {\varepsilon \left( {{\mathbf{q}},\omega } \right)}}} \right\}$, ${\text{Im}}\left\{ {{{ - 1} \mathord{\left/ {\vphantom {{ - 1} {\left[ {\varepsilon \left( {{\mathbf{q}}_{\parallel } ,\omega } \right) + 1} \right]}}} \right. \kern-0pt} {\left[ {\varepsilon \left( {{\mathbf{q}}_{\parallel } ,\omega } \right) + 1} \right]}}} \right\}$, respectively, where the complex quantity,$\varepsilon \left( {{\mathbf{q}},\omega } \right)$, is the bulk dielectric function of the material. $\hbar {\mathbf{q}}$ is the momentum transfer and $\hbar \omega$ is the energy loss of electrons that corresponds to the photon energy in an optical measurement. It is worth to mention that in both quantum and semi-classical approaches there is a quantitative difference in the intensity of surface excitations depending on the surface crossing direction. Experimentally it has been found that the difference becomes more obvious when an electron moves in a direction closer to the surface parallel^33^.

The RMC method combines the Monte Carlo simulation of electron scattering with a MCMC calculation of the parameterized optical ELF, ${\text{Im}}\left\{ {{{ - 1} \mathord{\left/ {\vphantom {{ - 1} {\varepsilon \left( \omega \right)}}} \right. \kern-0pt} {\varepsilon \left( \omega \right)}}} \right\}$. The *q*-dependent ELF, ${\text{Im}}\left\{ {{{ - 1} \mathord{\left/ {\vphantom {{ - 1} {\varepsilon \left( {q,\omega } \right)}}} \right. \kern-0pt} {\varepsilon \left( {q,\omega } \right)}}} \right\}$ is derived by employing Ritchie and Howie’s extrapolation scheme^25,34^ using $\omega_{q}^{2} (q,\omega_{{\text{p}}} ) = \omega_{{\text{p}}}^{2} + {{2E_{{\text{F}}} q^{2} } \mathord{\left/ {\vphantom {{2E_{{\text{F}}} q^{2} } 3}} \right. \kern-0pt} 3} + {{q^{4} } \mathord{\left/ {\vphantom {{q^{4} } 4}} \right. \kern-0pt} 4}$ as the plasmon dispersion relation, where $\omega_{q}$ and $\omega_{{\text{p}}}$ are the extended and original energy loss, respectively, and $E_{{\text{F}}}$ is the Fermi energy. The purpose of RMC procedure is to find the best ELF with the minimum difference between the simulated and experimental REELS according to the simulated annealing method^35^, which is a global optimization searching technique. Use a trial ELF composed by superimposing *N* Drude–Lindhard oscillators as input, then the Monte Carlo simulation is performed to produce a simulated REELS spectrum, $I^{{{\text{sim}}}} (\Delta E)$, where $\Delta E$ represents electron energy loss. Then an effective potential energy for the *n*th iteration, which represents the deviation between the simulated and experimental energy spectra as calculated by the weighted least square method, is defined as,

5 $$\chi_{n}^{2} = \mathop \sum \limits_{j} {{\left[ {I_{n}^{{\text{sim}}} (\Delta E_{j} ) - I_{n}^{\exp } (\Delta E_{j} )} \right]^{2} } \mathord{\left/ {\vphantom {{\left[ {I_{n}^{{\text{sim}}} (\Delta E_{j} ) - I_{n}^{\exp } (\Delta E_{j} )} \right]^{2} } {\sigma (\Delta E_{j} )^{2} }}} \right. \kern-0pt} {\sigma (\Delta E_{j} )^{2} }},$$

where $I_{n}^{\exp } (\Delta E_{j} )$ is the normalized experimental REELS spectrum with elastic peak area, and $\sigma (\Delta E_{j} )$ is a weighting factor to emphasizes the importance of the energy loss zone. In other words, the RMC method is an optimization of ELF by minimizing $\chi_{n}^{2}$ in a simulated annealing process. The final ELF will be obtained when $\chi_{n}^{2}$ converges.

The normalization procedure does not affect the obtained absolute ELF value; this is because the absolute ELF value affects the inelastic scattering cross section and, hence, the ratio of elastic scattering to inelastic scattering events. Once the experimental spectrum is known, the intensity of elastic peak (either measured in area or height) to inelastic peak then informs such ratio of scattering cross section between elastic scattering to inelastic scattering. This ELF obtained can reveal some optical properties or the electronic basic transition properties of the materials that people are interested in.

### Analysis of optical property

In the RMC procedure, the ELF to be solved can be described by a sum of *N* Drude–Lindhard terms containing 3*N* oscillator parameters:

6 $${\text{Im}}\left\{ {\frac{ - 1}{{\varepsilon \left( {q,\omega } \right)}}} \right\} = \sum\limits_{i = 1}^{N} {A_{i} } {\kern 1pt} {\text{Im}}\left\{ {\frac{ - 1}{{\varepsilon \left( {q,\omega ;\omega_{{{\text{p}}i}} ,\gamma_{i} } \right)}}} \right\},$$

where $A_{i}$, $\omega_{{{\text{p}}i}}$, $\gamma_{i}$ are the oscillator strength, energy and the width of the *i*th oscillator, respectively. The optical dielectric function $\varepsilon \left( \omega \right)$ refers to the long wavelength limit, $q \to 0$. After obtaining the optimal ELF by the RMC method, the derivation of the real part, ${\text{Re}}\left\{ {{{ - 1} \mathord{\left/ {\vphantom {{ - 1} {\varepsilon \left( \omega \right)}}} \right. \kern-0pt} {\varepsilon \left( \omega \right)}}} \right\}$, can be determined by an analytical Kramers–Kronig relation. Then the two parts of the dielectric function are:

7 $$\begin{aligned} \varepsilon_{1} = & \frac{{ - {\text{Re}} \left\{ {{{ - 1} \mathord{\left/ {\vphantom {{ - 1} {\varepsilon \left( \omega \right)}}} \right. \kern-0pt} {\varepsilon \left( \omega \right)}}} \right\}}}{{{\text{Im}} \left\{ {{{ - 1} \mathord{\left/ {\vphantom {{ - 1} {\varepsilon \left( \omega \right)}}} \right. \kern-0pt} {\varepsilon \left( \omega \right)}}} \right\}^{2} + {\text{Re}} \left\{ {{{ - 1} \mathord{\left/ {\vphantom {{ - 1} {\varepsilon \left( \omega \right)}}} \right. \kern-0pt} {\varepsilon \left( \omega \right)}}} \right\}^{2} }}, \\ \varepsilon_{2} = & \frac{{{\text{Im}} \left\{ {{{ - 1} \mathord{\left/ {\vphantom {{ - 1} {\varepsilon \left( \omega \right)}}} \right. \kern-0pt} {\varepsilon \left( \omega \right)}}} \right\}}}{{{\text{Im}} \left\{ {{{ - 1} \mathord{\left/ {\vphantom {{ - 1} {\varepsilon \left( \omega \right)}}} \right. \kern-0pt} {\varepsilon \left( \omega \right)}}} \right\}^{2} + {\text{Re}} \left\{ {{{ - 1} \mathord{\left/ {\vphantom {{ - 1} {\varepsilon \left( \omega \right)}}} \right. \kern-0pt} {\varepsilon \left( \omega \right)}}} \right\}^{2} }}. \\ \end{aligned}$$

And the refractive index $n$ and extinction coefficient $k$ can therefore be derived as:

8 $$\begin{aligned} n = & \sqrt {\frac{{\varepsilon_{1} + \sqrt {\varepsilon_{1}^{2} + \varepsilon_{2}^{2} } }}{2}} , \\ k = & \sqrt {\frac{{ - \varepsilon_{1} + \sqrt {\varepsilon_{1}^{2} + \varepsilon_{2}^{2} } }}{2}} . \\ \end{aligned}$$

### Sum rules

The reliability of the calculated ELF, optical constants and dielectric function extracted from REELS spectra can be checked by the various sum rules described below.

#### Oscillator-strength sum rule and perfect-screening sum rule

More widely used sum rules are the oscillator-strength sum rule (*f*-sum rule) and perfect-screening sum rule (*ps*-sum rule)^18^. The *f*-sum rule for optical ELF, ${\text{Im}}\left\{ {{{ - 1} \mathord{\left/ {\vphantom {{ - 1} {\varepsilon \left( \omega \right)}}} \right. \kern-0pt} {\varepsilon \left( \omega \right)}}} \right\}$, the extinction coefficient, $k$, and the imaginary part of the dielectric function, $\varepsilon_{2}$, are respectively defined as^18,25^:

9 $$Z_{{{\text{eff}}}} |_{{{\text{ELF}}}} = \frac{2}{{\pi {\kern 1pt} \Omega_{{\text{p}}}^{2} }}\int_{0}^{\infty } {\omega {\kern 1pt} {\text{Im}}\left\{ {{{ - 1} \mathord{\left/ {\vphantom {{ - 1} {\varepsilon \left( \omega \right)}}} \right. \kern-0pt} {\varepsilon \left( \omega \right)}}} \right\}{\kern 1pt} {\kern 1pt} } {\text{d}}\omega ,$$

10 $$Z_{{{\text{eff}}}} |_{k} = \frac{4}{{\pi {\kern 1pt} \Omega_{{\text{p}}}^{2} }}\int_{0}^{\infty } {\omega {\kern 1pt} k\left( \omega \right)} {\kern 1pt} {\text{d}}\omega ,$$

11 $$Z_{{{\text{eff}}}} |_{{\varepsilon_{2} }} = \frac{2}{{\pi {\kern 1pt} \Omega_{{\text{p}}}^{2} }}\int_{0}^{\infty } {\omega {\kern 1pt} \varepsilon_{2} \left( \omega \right){\kern 1pt} {\kern 1pt} } {\text{d}}\omega ,$$

where $\Omega_{p} = \sqrt {4\pi n_{a} }$ and $n_{a}$ is the atomic density of the sample. The *ps*-sum rule derived from Kramers–Kronig relations can be expressed by^25^

12 $$P_{{{\text{eff}}}} |_{{{\text{ELF}}}} = \frac{2}{\pi }\int_{0}^{\infty } {\frac{1}{\omega }{\text{Im}}\left\{ {{{ - 1} \mathord{\left/ {\vphantom {{ - 1} {\varepsilon \left( \omega \right)}}} \right. \kern-0pt} {\varepsilon \left( \omega \right)}}} \right\}{\kern 1pt} } {\kern 1pt} {\text{d}}\omega + {\text{Re}} \left\{ {{{ - 1} \mathord{\left/ {\vphantom {{ - 1} {\varepsilon \left( 0 \right)}}} \right. \kern-0pt} {\varepsilon \left( 0 \right)}}} \right\}.$$

For conductors, ${\text{Re}} \left\{ {{{ - 1} \mathord{\left/ {\vphantom {{ - 1} {\varepsilon \left( 0 \right)}}} \right. \kern-0pt} {\varepsilon \left( 0 \right)}}} \right\}$ is zero. The theoretical values for *f*- and *ps*-sum rules are the atomic number (i.e. $Z = 26$ for Fe and $Z = 28$ for Ni) and 1, respectively.

#### Inertial sum rule

Sum rule for testing refractive index $n\left( \omega \right)$, which is called inertial sum rule, is written as^25^,

13 $$R_{n} \left( \omega \right) = \int_{0}^{\omega } {\left[ {n\left( {\omega^{\prime}} \right) - 1} \right]{\text{d}}\omega^{\prime}} .$$

Since the theoretical value is $R_{n} \left( \infty \right) = 0$, it is convenient to defining a verification parameter^25^ to replace the conventional relative error,

14 $$\xi_{n} = \frac{{\int_{0}^{\infty } {\left[ {n\left( \omega \right) - 1} \right]{\text{d}}\omega } }}{{\int_{0}^{\infty } {\left| {n\left( {\omega^{\prime}} \right) - 1} \right|{\text{d}}\omega } }}.$$

#### dc-conductivity sum rule

As for the real part of the dielectric function $\varepsilon_{1}$, it can be tested by the dc-conductivity sum rule^25^,

15 $$R_{{\varepsilon_{1} }} \left( \omega \right) = \int_{0}^{\omega } {\left[ {\varepsilon_{1} \left( {\omega^{\prime}} \right) - 1} \right]} {\kern 1pt} {\kern 1pt} {\text{d}}\omega^{\prime},$$

whose theoretical value for metal is $R_{{\varepsilon_{1} }} \left( \infty \right) = - 2\pi^{2} \sigma_{0}$, where $\sigma_{0}$ is the dc-conductivity.

## Results and discussion

**Figure 1:**
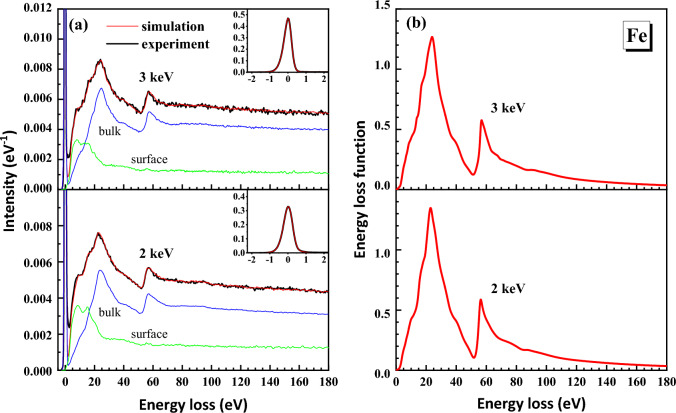
(**a**) The final simulated REELS spectra (red lines) and measured spectra (black lines) of Fe at 2 keV and 3 keV. Contributions from bulk and surface excitations from simulation are shown by the blue and green lines, respectively. The inset shows the elastic peak for convolution of the spectrum. (**b**) The corresponding ELFs of each energy obtained from the simulated REELS spectra.

**Figure 2:**
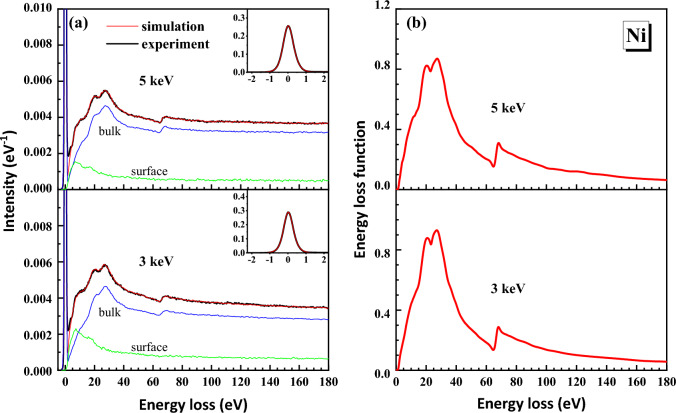
(**a**) The final simulated REELS spectra (red lines) and measured spectra (black lines) of Ni at 3 keV and 5 keV. Contributions from bulk and surface excitations from simulation are shown by the blue and green lines, respectively. The inset shows the elastic peak for convolution of the spectrum. (**b**) The corresponding ELFs of each energy obtained from the simulated REELS spectra.

The RMC method was applied to obtain the ELF of the samples from the measured REELS spectra. We found that the present ELFs of Fe and Ni show higher accuracy than the previous data^1,19,20,36^. The details of the reasons please see below. Figures 1a and 2a shows that excellent agreements between the simulated and the experimental REELS spectra were found for all the two primary energies and for the two elements. All REELS spectra are normalized with the area of the corresponding elastic peak which are shown in the inset of Figs. 1a and 2a. The ELFs obtained from the spectra are shown in Figs. 1b and 2b.

**Figure 3:**
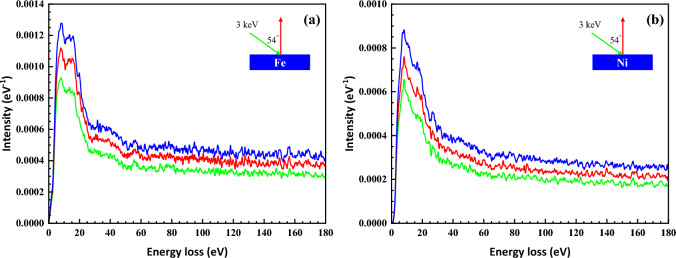
The simulated contributions to the surface component in REELS spectrum from electron trajectory parts for incoming to surface in vacuum (green lines), outgoing from surface in vacuum (red lines) and in solid (blue lines) in (**a**) Fe and (**b**) Ni for 3 keV primary electron beam and at 54° incident angle.

The surface contribution in DIIMFP actually consists three items as described in Eqs. (3) and (4): the incoming contribution in vacuum, the outgoing contribution in vacuum, and the contribution in the material. We have performed a calculation to discriminate the contributions to surface excitation from the three trajectory parts in order to show the accumulated effect of depth on surface excitation, taking Fe and Ni as examples and the primary electron energy of 3 keV, which are shown in Fig. 3. The bulk contribution and total surface contribution are provided in Figs. 1a and 2a.

**Figure 4:**
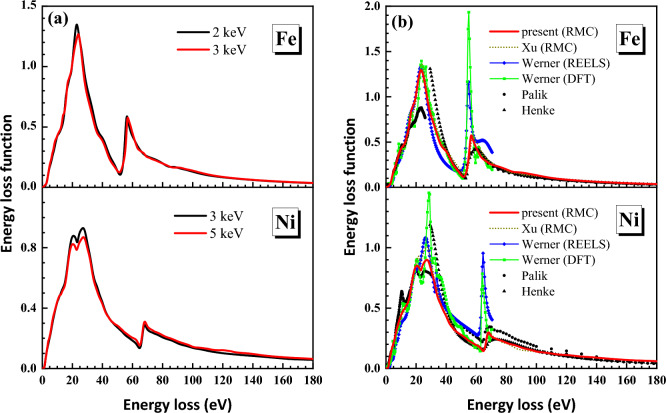
(**a**) Comparison of the ELFs for the two primary energies of Fe and Ni; (**b**) Comparison of ELFs deduced by the present RMC method (red lines) with the cutout data from Xu’s results (dotted lines)^19,20^, Werner’s REELS data (green lines)^15^, Werner’s DFT calculated data (blue lines)^15^, Palik’s compiled data (black circles)^1,2^ and Henke’s experiments data (black triangles)^36^ for Fe (Palik’s compiled data lack 1.5–5 eV and 26–50 eV energy loss regions) and Ni.

Figure 4a shows the comparison of the ELFs for the two primary energies. They agree with each other quite well. Figure 4b shows the averaged ELFs over two energies compared with Xu’s results^19,20^, with Werner’s results^15^ and with other data by optical methods^1,2,36^. Here we focus on the comparison with the previous REELS-RMC results^19,20^.

### Iron

**Figure 5:**
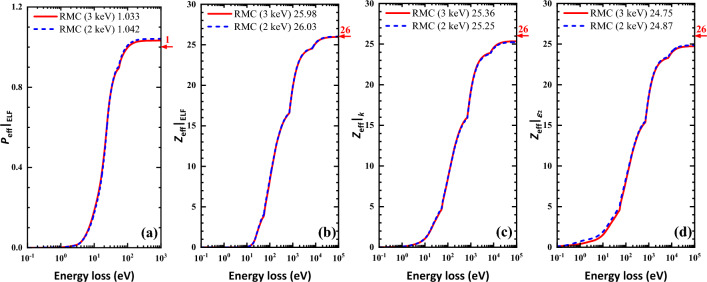
The *ps*- and *f*-sum rule checks for ELFs, the extinction coefficient, and the imaginary part of the dielectric function of Fe at the 2 keV (blue dashed lines) and 3 keV (red lines) primary energies whose theorical values are marked with red arrows and numbers on the right axes, and the calculated results were marked in the legend as well. (**a**) The *ps*-sum rule calculated by Eq. (12); (**b**) the *f*-sum rule calculated by Eq. (9); (**c**) the *f*-sum rule calculated by Eq. (10); (**d**) the *f*-sum rule calculated by Eq. (11).

As is shown in Fig. 4a, the final ELFs obtained from experimental REELS spectra at the primary energies of 2 keV and 3 keV are almost the same in the energy loss range of 0–180 eV. The difference is mainly existed at the first peak around 25 eV. And the averaged ELF of Fe in Fig. 4b is generally close to that of Xu et al. (the dotted line)^19^. In the low energy loss region (< 15 eV), the present and Xu’s RMC results agree well with Palik’s data. In the energy loss region below 25 eV, our RMC results are in agreement with Werner’s data obtained from a deconvolution of REELS spectra. We can also see from Fig. 4b that the RMC results are closer to Werner’s density functional theory (DFT) calculation data in the energy loss region about 25–50 eV. Meanwhile in the high energy loss region, ELFs determined from RMC method are closer to Henke’s data but have a sharper M_2,3_-edge around 55–60 eV. According to Eqs. (9, 10, 11, 12), the calculated *f*- and *ps*-sum rules data will converge at the high energy losses. These sum rules are used to check the accuracy of the ELF, the extinction coefficient and the imaginary part of the dielectric function (see Fig. 5). The sum rules with their relative errors are listed in Table 1 in comparison with the results of Xu et al.^19^.

**Table 1:** List of *f*-sum and *ps*-sum rules for Fe at 2 keV and 3 keV primary energies as compared with Xu et al.^19^.

	$Z_{{{\text{eff}}}} |_{{{\text{ELF}}}}$^a^	Relative error (%)	$P_{{{\text{eff}}}} |_{{{\text{ELF}}}}$^b^	Relative error (%)
2 keV				
Present	26.030	0.12	1.0420	4.20
Xu et al	25.80	− 0.76	1.045	4.5
3 keV				
Present	25.980	− 0.08	1.0333	3.33
Xu et al	26.24	0.92	1.051	5.1
Averaging				
Present	26.005	0.02	1.0377	3.77

^a^The theoretical nominal value for *f*-sum rule of ELF is the atomic number of iron, i.e. 26.

^b^The theoretical nominal value for *ps*-sum rule is unit.

The improvements of ELF derived from the experimental REELS spectra of Fe as compared with Xu et al.^19^ are highlighted from two aspects. Firstly, the ELF was derived in a wider energy loss range (0–180 eV rather than 0–100 eV) as is shown in Fig. 4b. Secondly, more accurate sum rules at each primary electron energy are obtained than previous Xu’s ELFs^19^. Particularly, the *f*-sum rule check for the averaged ELF is 26.005 with relative error of 0.02%, which is very satisfactory.

**Figure 6:**
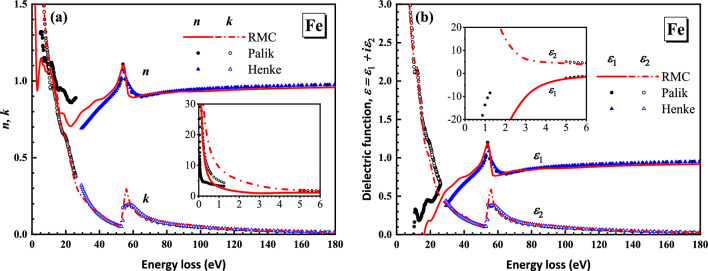
Comparison of (**a**) the refractive index *n* and the extinction coefficient *k* and (**b**) the real and imaginary parts of the complex dielectric function of Fe obtained from the present RMC method (red lines) with Palik’s (black circles)^2^ and Henke’s data (blue triangles)^36^.

Applying Eqs. (7) and (8), the optical constants, i.e., the refractive index *n* and the extinction coefficient *k*, and the complex dielectric function were calculated from the averaged ELF and shown in Fig. 6.

Similar to Xu’s work^19^, as is shown in Fig. 6a, our present data of refractive index and extinction coefficient are in good agreement with the experimental data in the high energy loss region, and our present data also smoothly join the extinction coefficient of Palik’s data in the absent range of 26–50 eV. Furthermore, we supplement the calculation results of the complex dielectric function in Fig. 6b, which also reasonably agree with Palik’s and Henke’s data. In addition, we also used Eqs. (13) and (15) to calculate the inertial sum rule and dc-conductivity sum rule: the result of $\xi_{n}$ is − 0.2179 and the relative error of $R_{{\varepsilon_{1} }} (\omega )$ is − 60.76%. The accuracy is limited by the absent information at the very low loss energies below 1 eV in the REELS spectra, which was blurred by the measured elastic peak broadening.

### Nickel

**Figure 7:**
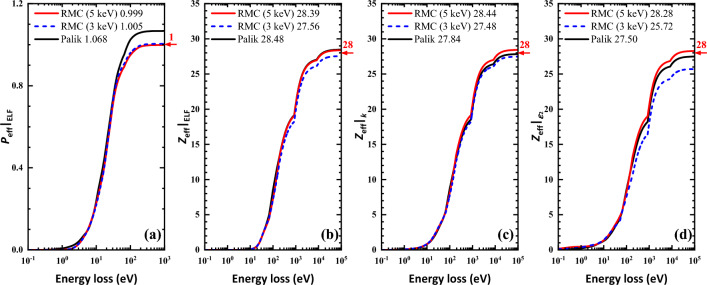
The *ps*- and *f*-sum rule checks for ELFs, the extinction coefficient, and the imaginary part of the dielectric function of Ni at the 3 keV (blue dashed lines) and 5 keV (red lines) primary energies whose theorical values are marked with red arrows and numbers on the right axes, and the calculated results were marked in the legend as well. (**a**) The *ps*-sum rule calculated by Eq. (12); (**b**) the *f*-sum rule calculated by Eq. (9); (**c**) the *f*-sum rule calculated by Eq. (10); (**d**) the *f*-sum rule calculated by Eq. (11).

In the case of Ni, the two ELFs obtained by the present RMC method in Fig. 4a were averaged and displayed in Fig. 4b in comparison with Xu’s results^20^, with Werner’s data^15^, with Palik’s data^1^, and with Henke’s experimental data^36^. In Palik’s database there is only a small amount of data, merely four data points, in the energy loss range of 30–50 eV. In the whole energy loss range shown, the present ELF is close to Xu’s ELF^20^ except the intermediate energy loss region around 20–30 eV where our present ELF has lower values and it is also weaker than that of Werner’s two datasets. Moreover, below 10 eV the five datasets, i.e. the present, Xu et al.^20^, Werner et al.^15^ and Palik^1^, are almost the same. In the high energy loss region above 50 eV, Werner’s ELFs have sharper peak at M_2,3_-edge than other datasets around 70 eV. The present and Xu’s ELF are close to Henke’s data but the first two datasets have sharper M_2,3_-edge. The *f*- and *ps*-sum rules were calculated to check the accuracy of the ELF, the extinction coefficient and the imaginary part of the dielectric function (see Fig. 7) at primary energies of 3 keV and 5 keV and compared with the Xu et al.^20^ and with the Palik’s results. The results with the relative errors in comparison with the results of Xu et al.^20^ are given in Table 2.

**Table 2:** List of *f*-sum and *ps*-sum rules for Ni at 3 keV and 5 keV primary energies as compared with Xu et al.^20^.

	$Z_{{{\text{eff}}}} |_{{{\text{ELF}}}}$^a^	Relative error (%)	$P_{{{\text{eff}}}} |_{{{\text{ELF}}}}$^b^	Relative error (%)
3 keV				
Present	27.560	− 1.57	1.0051	0.51
Xu et al	27.14	− 3.1	1.006	0.6
5 keV				
Present	28.389	1.39	0.9991	− 0.09
Averaging				
Present	27.974	− 0.09	1.0021	0.21

^a^The theoretical nominal value for *f*-sum rule of ELF is the atomic number of nickel, i.e. 28.

^b^The theoretical nominal value for *ps*-sum rule is unit.

One can see in Fig. 7a that our present RMC results perform much better than the Palik’s data for the *ps*-sum rule as the convergence values of RMC is closer to the theoretical value. From the results listed in Table 2, we can see that our present ELFs have been improved mainly in two aspects as compared with Xu’s results^20^. At 3 keV, our new ELF has better accuracy especially for $Z_{{{\text{eff}}}} |_{{{\text{ELF}}}}$, for which the relative error has been reduced by half. In addition, we derived the ELF from experimental REELS at primary electron energy of 5 keV that not included in Xu’s work^20^. The result of the higher primary energy presents much more accurate *ps*-sum rule. Moreover, the new averaged ELFs of 3 keV and 5 keV have very small relative errors of − 0.09% and 0.21% for $Z_{{{\text{eff}}}} |_{{{\text{ELF}}}}$ and $P_{{{\text{eff}}}} |_{{{\text{ELF}}}}$, respectively.

**Figure 8:**
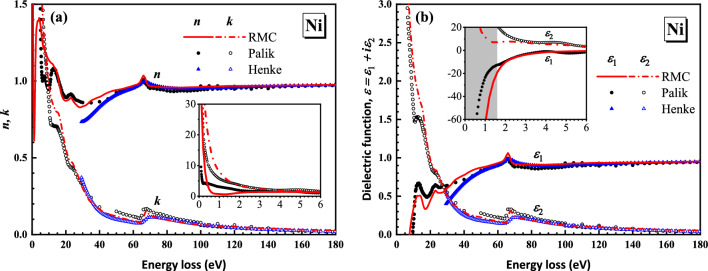
Comparison of (**a**) the refractive index *n* and the extinction coefficient *k* and (**b**) the real and imaginary parts of the complex dielectric function of Ni obtained from the present RMC method (red lines) with Palik’s (black circles)^1^ and Henke’s data (blue triangles)^36^.

Figure 8a shows the calculated optical constants of Ni by using the present averaged ELF in the photon energy range between 0 and 180 eV in comparison with Palik’s^1^ and Henke’s data^36^. In the higher energy loss region above the M_2,3_-edge, the three datasets finally converge. The difference is mainly found in the low energy loss region which can be predicted from Fig. 7a,b. The real and imaginary parts of dielectric function derived from the averaged ELF are compared with Palik’s^1^ and Henke’s data^36^ in Fig. 8b. As is shown in Fig. 8b, dielectric function from the present RMC method shows the same trend as others. Especially, in Xu’s work^20^ it was mentioned that the behavior of the RMC data in infrared region (below 1.6 eV) is not true for it conflicts with the prediction of Drude dielectric function model in the low frequency region, where $\varepsilon_{1} (\omega )$ should approach to negative infinity. In our present result, however, we get reasonable results in the infrared region shown by the shadow area in the inset of Fig. 8b, which has been improved in contrast to the results of Xu et al.^20^. Moreover, the inertial sum rule and the relative error of dc-conductivity sum rule have been calculated as − 0.1387 and − 94.12%, respectively, whose error source is the same as Fe.

## Conclusions

The RMC method provides a global optimization technique with a MCMC method to obtain inherent optical properties of metals, which combines the Mott’s cross section for elastic scattering and depth-dependent DIIMFP within a semi-classical framework for inelastic scattering. In this work, the improved ELFs compared with Xu^19,20^ of Fe and Ni have been derived from the measured REELS spectra with the RMC analysis. In the case of Fe, we have updated the values of ELFs as well as improved their accuracy justified with *f*- and *ps*-sum rules in a wider energy loss range of 0–180 eV at the primary electron energies of 2 keV and 3 keV. In the case of Ni, we not only improved the accuracy of ELF, whose relative errors of sum rules were reduced at primary energy of 3 keV in contrast to Xu^20^, but also supplemented the ELF at higher energy of 5 keV. Based on the new ELFs, the optical constants and dielectric function of Fe and Ni were calculated. Our results of *f*- and *ps*-sum rules are much closer to the nominal theoretical values than any other previous data. Due to the high accuracy of the present data (included in supplementary material) the use of our data is highly recommended for further applications in materials science.

## Aacknowledgements

This work was supported by the Fund of Science and Technology on Surface Physics and Chemistry Laboratory (XKFZ202103), Chinese Education Ministry through “111 Project 2.0” (BP0719016), the Fund for Bilateral Relationships between China and Hungary in Science and Technology (2021-1.2.4-TÉT-2021-00055), the Kurata Grants from The Hitachi Global Foundation and from The Iketani Science & Technology Foundation. We thank Prof. H.M. Li and the supercomputing center of USTC for the support of parallel computing.

## Supplementary Information

Supplementary Information.

## Data Availability

The datasets generated and/or analysed during the current study available from the corresponding author on reasonable request.
