# Supplementary material for: Improved reverse Monte Carlo analysis of optical property of Fe and Ni from reflection electron energy loss spectroscopy spectra
Source: Sci Rep. 2023 Aug 1;13:12480. doi: 10.1038/s41598-023-38769-4 (PMC10393999; doi:10.1038/s41598-023-38769-4)
Supplement: Supplementary file 1 — Supplementary Information. [file 41598_2023_38769_MOESM1_ESM.docx]

**Supplementary material**

**Table 1**. The oscillator parameters of averaged energy loss functions (ELFs) for Fe and Ni ($A_{i}$, $\omega_{pi}$ and $\gamma_{i}$ are the oscillator strength, energy and the width of the $i$th oscillator, respectively).

| Fe | | | | Ni | | | |
| --- | --- | --- | --- | --- | --- | --- | --- |
| $i$ | $A_{i}$ | $\omega_{pi}$ | $\gamma_{i}$ | $i$ | $A_{i}$ | $\omega_{pi}$ | $\gamma_{i}$ |
| 1 | -5.0700E-3 | 1.4040 | 2.0213 | 1 | 6.2440E-4 | 1.0025 | 0.4990 |
| 2 | -1.1900E-3 | 1.4388 | 3.8290 | 2 | -3.0068E-2 | 1.4411 | 2.0277 |
| 3 | -1.0000E-3 | 1.6040 | 1.0020 | 3 | -1.2593E-2 | 1.7411 | 1.4585 |
| 4 | -7.5400E-3 | 1.9559 | 3.5290 | 4 | -1.3493E-2 | 1.7773 | 1.4585 |
| 5 | -1.0300E-3 | 2.0000 | 1.0000 | 5 | -5.3391E-4 | 2.0928 | 0.8576 |
| 6 | 3.9100E-3 | 2.4920 | 1.8034 | 6 | -2.0339E-3 | 2.6928 | 0.8576 |
| 7 | -3.0000E-2 | 3.1544 | 2.8951 | 7 | 1.6044E-3 | 3.9825 | 0.8062 |
| 8 | -2.7500E-2 | 3.2530 | 3.1380 | 8 | -3.6044E-3 | 3.9825 | 3.1062 |
| 9 | 5.7100E-3 | 4.6940 | 2.7227 | 9 | -1.6044E-3 | 4.0825 | 2.1062 |
| 10 | 5.5800E-3 | 5.1561 | 2.2586 | 10 | -1.5890E-2 | 6.0828 | 3.2750 |
| 11 | 2.0000E-3 | 6.7354 | 1.3280 | 11 | 6.6530E-3 | 6.5661 | 2.8255 |
| 12 | 1.1000E-3 | 6.9578 | 1.3280 | 12 | 3.5265E-3 | 7.0661 | 2.8255 |
| 13 | 7.4800E-3 | 8.4716 | 2.3687 | 13 | -1.1760E-4 | 10.7000 | 0.5797 |
| 14 | 1.4200E-3 | 8.4862 | 2.3687 | 14 | -2.5000E-3 | 12.0299 | 3.0025 |
| 15 | 2.0140E-2 | 9.6592 | 10.5510 | 15 | -1.6459E-3 | 12.5299 | 1.9025 |
| 16 | 1.6980E-2 | 10.0935 | 4.0560 | 16 | 5.4806E-4 | 13.8000 | 4.2962 |
| 17 | 1.1780E-2 | 10.7410 | 4.3295 | 17 | 5.0000E-4 | 14.0299 | 2.4507 |
| 18 | 2.8110E-2 | 10.9363 | 11.4506 | 18 | 9.8064E-5 | 14.1000 | 3.3962 |
| 19 | 3.8700E-3 | 12.3864 | 3.5410 | 19 | -8.1096E-2 | 15.5799 | 6.4481 |
| 20 | 3.2500E-3 | 13.7679 | 1.5187 | 20 | 7.0481E-3 | 16.1799 | 6.4481 |
| 21 | 6.8600E-3 | 15.6890 | 2.2600 | 21 | 1.8784E-2 | 18.8214 | 7.3250 |
| 22 | 1.5100E-3 | 16.5880 | 1.6220 | 22 | -9.8435E-5 | 19.3491 | 6.5117 |
| 23 | 9.4700E-3 | 16.9145 | 2.3128 | 23 | 1.9575E-2 | 19.8439 | 6.5561 |
| 24 | -3.6834E-4 | 16.9845 | 1.3128 | 24 | 7.7844E-4 | 19.8491 | 5.0117 |
| 25 | -3.2100E-3 | 17.3145 | 2.3028 | 25 | -3.6082E-3 | 22.9473 | 2.6634 |
| 26 | 2.0500E-2 | 17.8270 | 4.5720 | 26 | -3.0482E-3 | 23.3121 | 2.1096 |
| 27 | 2.9530E-2 | 18.0190 | 5.5537 | 27 | 8.0466E-1 | 23.8512 | 31.2539 |
| 28 | -1.0400E-3 | 19.0924 | 1.9163 | 28 | 1.5036E-2 | 24.4330 | 9.1792 |
| 29 | 7.5000E-4 | 19.3000 | 1.5000 | 29 | 1.5036E-2 | 24.6330 | 7.3792 |
| 30 | -2.6600E-3 | 19.3448 | 1.7690 | 30 | -2.2255E-4 | 26.2073 | 2.1396 |
| 31 | 3.0830E-2 | 21.0676 | 7.4643 | 31 | 1.3843E-2 | 27.4073 | 6.0358 |
| 32 | 4.3000E-2 | 21.6045 | 6.6138 | 32 | 1.9122E-2 | 27.4073 | 6.3417 |
| 33 | 5.5310E-2 | 22.9442 | 6.3220 | 33 | 1.2480E-2 | 28.7783 | 4.8007 |
| 34 | 9.4500E-3 | 23.0297 | 3.5987 | 34 | 2.6160E-2 | 31.5154 | 6.2104 |
| 35 | 3.1080E-2 | 23.4835 | 7.3936 | 35 | 9.1785E-4 | 32.2620 | 3.6448 |
| 36 | -5.4759E-4 | 24.1297 | 3.0000 | 36 | 9.1785E-4 | 32.8164 | 3.6743 |
| 37 | 1.1010E-2 | 24.1587 | 3.4060 | 37 | 1.7080E-2 | 35.0069 | 19.5932 |
| 38 | 2.0600E-2 | 25.8368 | 5.1316 | 38 | 1.2874E-3 | 35.7219 | 10.1382 |
| 39 | 2.2880E-2 | 26.0610 | 5.5187 | 39 | 5.3200E-3 | 36.0502 | 4.7834 |
| 40 | 2.9000E-3 | 36.0406 | 12.4613 | 40 | 5.4230E-4 | 36.5912 | 3.1382 |
| 41 | -2.3730E-2 | 36.3981 | 9.2701 | 41 | 2.8125E-4 | 37.4583 | 2.6583 |
| 42 | -2.5540E-2 | 36.8520 | 8.5130 | 42 | 9.8200E-3 | 39.7205 | 6.1969 |
| 43 | 7.5000E-4 | 37.0090 | 3.0000 | 43 | 3.4466E-3 | 41.9705 | 21.5776 |
| 44 | 3.2400E-1 | 37.4790 | 39.9370 | 44 | 7.5000E-4 | 44.0000 | 5.0000 |
| 45 | 3.3007E-1 | 37.8827 | 44.0329 | 45 | 9.2693E-4 | 44.0000 | 4.9461 |
| 46 | 1.0000E-3 | 38.0270 | 12.8310 | 46 | 1.2672E-3 | 45.0275 | 6.9280 |
| 47 | -5.4101E-4 | 43.0970 | 228.2277 | 47 | 5.0000E-4 | 46.9581 | 3.3905 |
| 48 | -4.0283E-4 | 44.4301 | 5.3527 | 48 | 5.3305E-4 | 49.2643 | 10.8821 |
| 49 | -2.9020E-2 | 45.2500 | 13.1081 | 49 | 5.3305E-4 | 49.2643 | 11.3354 |
| 50 | -3.4250E-2 | 45.2500 | 13.4720 | 50 | 5.5176E-4 | 50.4335 | 5.9702 |
| 51 | -3.7800E-2 | 53.2670 | 10.5662 | 51 | 1.5176E-4 | 50.4335 | 8.9702 |
| 52 | 2.6800E-2 | 54.2421 | 6.9504 | 52 | 3.4073E-3 | 50.4774 | 9.9868 |
| 53 | -6.6130E-2 | 54.3915 | 10.4017 | 53 | 3.4073E-3 | 50.4774 | 9.3235 |
| 54 | 1.0140E-2 | 56.0200 | 3.2000 | 54 | 3.5351E-4 | 53.2435 | 2.9702 |
| 55 | 3.8400E-3 | 56.5223 | 2.0056 | 56 | 1.7894E-4 | 55.7100 | 3.2754 |
| 56 | 5.6500E-3 | 57.4000 | 3.3858 | 57 | 1.8156E-4 | 56.9205 | 11.7944 |
| 57 | 8.9698E-4 | 57.5066 | 3.4790 | 58 | 3.3894E-4 | 56.9205 | 12.1754 |
| 58 | 5.1500E-3 | 58.2066 | 6.0790 | 59 | 1.3942E-3 | 58.0015 | 7.9506 |
| 59 | 2.7300E-3 | 58.6351 | 3.4790 | 60 | -5.8267E-4 | 60.5138 | 7.9636 |
| 60 | -2.6830E-5 | 59.5535 | 1.0562 | 61 | -1.0827E-3 | 60.5138 | 8.9636 |
| 61 | 5.0700E-3 | 59.9122 | 5.4124 | 62 | -4.5095E-4 | 61.8038 | 10.6682 |
| 62 | -1.0500E-3 | 63.3544 | 5.2990 | 63 | 5.5274E-4 | 62.6441 | 5.0303 |
| 63 | -5.5346E-4 | 63.4094 | 3.0000 | 64 | 3.9774E-4 | 62.6441 | 6.7928 |
| 64 | 3.3000E-2 | 64.1044 | 15.2990 | 65 | 2.2774E-4 | 63.8000 | 3.0303 |
| 65 | 3.0210E-2 | 64.3750 | 16.8607 | 66 | -3.4986E-3 | 64.9982 | 5.0147 |
| 66 | -1.7540E-2 | 65.7350 | 12.4530 | 67 | -4.6736E-3 | 65.4882 | 6.0582 |
| 67 | 2.4800E-3 | 67.7064 | 5.0000 | 68 | -3.0078E-3 | 66.2055 | 5.2501 |
| 68 | 4.8600E-3 | 69.3683 | 16.7051 | 69 | -3.0078E-3 | 66.2055 | 5.4689 |
| 69 | -3.7500E-2 | 69.5557 | 13.8741 | 70 | 5.6390E-3 | 67.5068 | 4.3662 |
| 70 | 1.5000E-3 | 70.4280 | 18.2380 | 71 | 5.5652E-3 | 67.6068 | 4.5638 |
| 71 | 4.9500E-3 | 70.7750 | 6.8957 | 72 | 1.7620E-2 | 67.9814 | 17.4489 |
| 72 | 1.3360E-2 | 71.0782 | 18.5172 | 73 | 2.0578E-4 | 69.3375 | 3.8405 |
| 73 | -1.9000E-2 | 71.5400 | 21.9230 | 74 | 3.0151E-4 | 71.0289 | 3.6646 |
| 74 | 1.0000E-3 | 72.1680 | 18.9890 | 75 | 3.0039E-4 | 71.3375 | 3.5405 |
| 75 | -2.1140E-2 | 73.4771 | 24.6448 | 76 | 2.5651E-4 | 73.0289 | 3.6646 |
| 76 | 4.2500E-3 | 73.7565 | 15.2266 | 77 | 2.7651E-4 | 73.0289 | 3.5499 |
| 77 | 9.8000E-3 | 74.1517 | 21.3391 | 78 | 4.0700E-3 | 75.7289 | 9.5646 |
| 78 | 2.0000E-3 | 74.1570 | 15.5820 | 79 | 1.2847E-3 | 78.8210 | 11.5646 |
| 79 | 7.5000E-4 | 74.3500 | 7.0000 | 80 | 1.6423E-3 | 78.8210 | 10.5646 |
| 80 | 4.6430E-2 | 75.4480 | 48.8484 | 81 | 7.9500E-3 | 85.8424 | 16.2295 |
| 81 | 5.3922E-4 | 79.3172 | 173.3280 | 82 | 9.4358E-4 | 91.5875 | 158.6942 |
| 82 | 8.5000E-3 | 81.1426 | 173.3280 | 83 | -2.5000E-4 | 91.7578 | 8.4024 |
| 83 | -5.5000E-3 | 81.5070 | 34.9208 | 84 | -5.0001E-4 | 92.2797 | 16.2150 |
| 84 | 1.0000E-3 | 81.6318 | 9.5220 | 85 | -5.0000E-5 | 95.4578 | 4.0024 |
| 85 | 1.0000E-3 | 82.2142 | 33.1960 | 86 | -7.5009E-5 | 97.0254 | 5.8213 |
| 86 | -7.4000E-3 | 84.2098 | 14.6370 | 87 | 4.0400E-3 | 98.2638 | 21.3100 |
| 87 | -1.4400E-3 | 86.3508 | 10.0000 | 88 | 3.4745E-4 | 99.0885 | 258.4947 |
| 88 | 2.0500E-3 | 87.2300 | 10.5280 | 89 | 4.7600E-3 | 99.9108 | 18.9565 |
| 89 | 2.6870E-2 | 87.6869 | 48.8484 | 90 | 8.9266E-4 | 99.9994 | 22.4476 |
| 90 | -9.5945E-5 | 89.5300 | 6.0280 | 91 | 2.0942E-5 | 100.0000 | 10.8399 |
| 91 | -1.9595E-4 | 91.5000 | 10.0280 | 92 | 1.9086E-4 | 100.0000 | 8.9277 |
| 92 | 5.5178E-4 | 93.0773 | 7.9280 | 93 | -7.5398E-4 | 100.0000 | 29.6225 |
| 93 | -9.2175E-4 | 94.0857 | 9.4874 | 94 | 6.0898E-4 | 100.0000 | 24.2678 |
| 94 | 6.9500E-2 | 94.7720 | 230.6930 | 95 | -2.7600E-3 | 100.5000 | 10.3474 |
| 95 | 1.0000E-3 | 96.7610 | 94.9040 | 96 | 5.5234E-5 | 110.0500 | 4.5001 |
| 96 | 1.0000E-3 | 97.2020 | 23.6600 | 97 | 3.5222E-4 | 121.0000 | 9.5063 |
| 97 | 1.0600E-3 | 97.3669 | 22.6113 | 98 | 5.2222E-4 | 122.0000 | 24.1201 |
| 98 | 4.3857E-4 | 97.6375 | 94.1334 | 99 | 5.2421E-5 | 125.3000 | 4.1201 |
| 99 | 3.3900E-3 | 100.7560 | 53.5644 | 100 | 2.2194E-4 | 135.5000 | 15.0001 |
| 100 | 3.5000E-3 | 100.7560 | 55.8370 | 101 | 3.8889E-4 | 140.0000 | 20.1163 |
| 101 | 6.8700E-2 | 101.5668 | 230.6930 | 102 | 1.2128E-1 | 148.8534 | 256.0724 |
| 102 | -1.7180E-2 | 103.9190 | 51.8020 | 103 | -4.4524E-4 | 165.0000 | 25.5398 |
| 104 | 5.0000E-6 | 106.1210 | 4.5030 | 104 | 2.5499E-4 | 180.0000 | 30.3427 |
| 105 | 5.0000E-5 | 110.8630 | 123.7040 | 105 | 4.7879E-5 | 672.1765 | 146.9890 |
| 106 | 5.5000E-4 | 125.4940 | 43.6360 |  |  |  |  |

**Table 2**. Values of energy loss functions (ELFs) of Fe and Ni calculated from RMC.

| E (eV) | ELF | |
| --- | --- | --- |
|  | Fe | Ni |
| 0.5 | 0.00205 | 0.00080 |
| 1.0 | 0.00590 | 0.00275 |
| 1.5 | 0.01111 | 0.01234 |
| 2.0 | 0.01762 | 0.04643 |
| 2.5 | 0.02643 | 0.08222 |
| 3.0 | 0.04361 | 0.11417 |
| 3.5 | 0.07494 | 0.14338 |
| 4.0 | 0.11253 | 0.17159 |
| 4.5 | 0.14849 | 0.19014 |
| 5.0 | 0.17848 | 0.21151 |
| 5.5 | 0.20219 | 0.23529 |
| 6.0 | 0.22606 | 0.26334 |
| 6.5 | 0.25557 | 0.29381 |
| 7.0 | 0.28281 | 0.32124 |
| 7.5 | 0.30694 | 0.34434 |
| 8.0 | 0.33735 | 0.36537 |
| 8.5 | 0.36626 | 0.38579 |
| 9.0 | 0.38795 | 0.40569 |
| 9.5 | 0.40729 | 0.42465 |
| 10.0 | 0.42505 | 0.44208 |
| 10.5 | 0.43901 | 0.45654 |
| 11.0 | 0.44984 | 0.47004 |
| 11.5 | 0.46057 | 0.48098 |
| 12.0 | 0.47369 | 0.48910 |
| 12.5 | 0.49075 | 0.49793 |
| 13.0 | 0.51475 | 0.51033 |
| 13.5 | 0.54860 | 0.52214 |
| 14.0 | 0.57813 | 0.53205 |
| 14.5 | 0.60615 | 0.54287 |
| 15.0 | 0.65132 | 0.55892 |
| 15.5 | 0.70921 | 0.58309 |
| 16.0 | 0.76695 | 0.61536 |
| 16.5 | 0.82140 | 0.65324 |
| 17.0 | 0.85841 | 0.69328 |
| 17.5 | 0.88196 | 0.73226 |
| 18.0 | 0.90420 | 0.76769 |
| 18.5 | 0.92425 | 0.79777 |
| 19.0 | 0.94510 | 0.82132 |
| 19.5 | 0.98008 | 0.83775 |
| 20.0 | 1.03099 | 0.84728 |
| 20.5 | 1.08583 | 0.85087 |
| 21.0 | 1.13971 | 0.84969 |
| 21.5 | 1.19050 | 0.84439 |
| 22.0 | 1.23548 | 0.83484 |
| 22.5 | 1.27056 | 0.82183 |
| 23.0 | 1.29062 | 0.81247 |
| 23.5 | 1.29307 | 0.81977 |
| 24.0 | 1.27907 | 0.83992 |
| 24.5 | 1.25117 | 0.85795 |
| 25.0 | 1.21456 | 0.87082 |
| 25.5 | 1.17328 | 0.88041 |
| 26.0 | 1.12691 | 0.88831 |
| 26.5 | 1.07512 | 0.89515 |
| 27.0 | 1.02088 | 0.89938 |
| 27.5 | 0.96856 | 0.89862 |
| 28.0 | 0.92120 | 0.89166 |
| 28.5 | 0.87980 | 0.87816 |
| 29.0 | 0.84395 | 0.85887 |
| 29.5 | 0.81259 | 0.83621 |
| 30.0 | 0.78454 | 0.81319 |
| 30.5 | 0.75868 | 0.79126 |
| 31.0 | 0.73405 | 0.76967 |
| 31.5 | 0.70987 | 0.74657 |
| 32.0 | 0.68551 | 0.72058 |
| 32.5 | 0.66053 | 0.69159 |
| 33.0 | 0.63468 | 0.66109 |
| 33.5 | 0.60796 | 0.63170 |
| 34.0 | 0.58066 | 0.60582 |
| 34.5 | 0.55343 | 0.58452 |
| 35.0 | 0.52732 | 0.56749 |
| 35.5 | 0.50358 | 0.55329 |
| 36.0 | 0.48341 | 0.53992 |
| 36.5 | 0.46725 | 0.52580 |
| 37.0 | 0.45412 | 0.51070 |
| 37.5 | 0.44270 | 0.49559 |
| 38.0 | 0.43281 | 0.48145 |
| 38.5 | 0.42426 | 0.46889 |
| 39.0 | 0.41611 | 0.45735 |
| 39.5 | 0.40734 | 0.44552 |
| 40.0 | 0.39722 | 0.43245 |
| 40.5 | 0.38539 | 0.41817 |
| 41.0 | 0.37180 | 0.40349 |
| 41.5 | 0.35662 | 0.38947 |
| 42.0 | 0.34019 | 0.37688 |
| 42.5 | 0.32297 | 0.36607 |
| 43.0 | 0.30548 | 0.35687 |
| 43.5 | 0.28828 | 0.34875 |
| 44.0 | 0.27192 | 0.34104 |
| 44.5 | 0.25683 | 0.33338 |
| 45.0 | 0.24321 | 0.32591 |
| 45.5 | 0.23101 | 0.31909 |
| 46.0 | 0.21999 | 0.31324 |
| 46.5 | 0.20978 | 0.30822 |
| 47.0 | 0.19997 | 0.30338 |
| 47.5 | 0.19015 | 0.29834 |
| 48.0 | 0.18000 | 0.29347 |
| 48.5 | 0.16932 | 0.28915 |
| 49.0 | 0.15810 | 0.28534 |
| 49.5 | 0.14663 | 0.28172 |
| 50.0 | 0.13548 | 0.27793 |
| 50.5 | 0.12562 | 0.27375 |
| 51.0 | 0.11840 | 0.26915 |
| 51.5 | 0.11547 | 0.26432 |
| 52.0 | 0.11858 | 0.25960 |
| 52.5 | 0.12930 | 0.25518 |
| 53.0 | 0.14877 | 0.25076 |
| 53.5 | 0.17781 | 0.24561 |
| 54.0 | 0.21766 | 0.23994 |
| 54.5 | 0.27095 | 0.23470 |
| 55.0 | 0.34119 | 0.23027 |
| 55.5 | 0.42741 | 0.22639 |
| 56.0 | 0.51522 | 0.22257 |
| 56.5 | 0.56989 | 0.21869 |
| 57.0 | 0.56539 | 0.21490 |
| 57.5 | 0.53963 | 0.21125 |
| 58.0 | 0.51588 | 0.20767 |
| 58.5 | 0.49480 | 0.20411 |
| 59.0 | 0.47439 | 0.20059 |
| 59.5 | 0.45398 | 0.19719 |
| 60.0 | 0.43608 | 0.19398 |
| 60.5 | 0.41919 | 0.19096 |
| 61.0 | 0.40273 | 0.18797 |
| 61.5 | 0.38675 | 0.18470 |
| 62.0 | 0.37117 | 0.18065 |
| 62.5 | 0.35592 | 0.17530 |
| 63.0 | 0.34161 | 0.16834 |
| 63.5 | 0.32983 | 0.15990 |
| 64.0 | 0.32116 | 0.15102 |
| 64.5 | 0.31407 | 0.14477 |
| 65.0 | 0.30723 | 0.14593 |
| 65.5 | 0.30052 | 0.15869 |
| 66.0 | 0.29427 | 0.18452 |
| 66.5 | 0.28869 | 0.22052 |
| 67.0 | 0.28353 | 0.25834 |
| 67.5 | 0.27812 | 0.28661 |
| 68.0 | 0.27203 | 0.29831 |
| 68.5 | 0.26564 | 0.29577 |
| 69.0 | 0.25991 | 0.28639 |
| 69.5 | 0.25554 | 0.27612 |
| 70.0 | 0.25249 | 0.26773 |
| 70.5 | 0.25016 | 0.26183 |
| 71.0 | 0.24790 | 0.25769 |
| 71.5 | 0.24538 | 0.25427 |
| 72.0 | 0.24264 | 0.25131 |
| 72.5 | 0.23990 | 0.24898 |
| 73.0 | 0.23735 | 0.24680 |
| 73.5 | 0.23502 | 0.24413 |
| 74.0 | 0.23280 | 0.24123 |
| 74.5 | 0.23053 | 0.23864 |
| 75.0 | 0.22812 | 0.23649 |
| 75.5 | 0.22552 | 0.23462 |
| 76.0 | 0.22275 | 0.23276 |
| 76.5 | 0.21986 | 0.23077 |
| 77.0 | 0.21690 | 0.22858 |
| 77.5 | 0.21389 | 0.22620 |
| 78.0 | 0.21084 | 0.22367 |
| 78.5 | 0.20776 | 0.22104 |
| 79.0 | 0.20465 | 0.21837 |
| 79.5 | 0.20148 | 0.21571 |
| 80.0 | 0.19827 | 0.21311 |
| 80.5 | 0.19499 | 0.21063 |
| 81.0 | 0.19166 | 0.20831 |
| 81.5 | 0.18829 | 0.20616 |
| 82.0 | 0.18495 | 0.20421 |
| 82.5 | 0.18169 | 0.20242 |
| 83.0 | 0.17863 | 0.20077 |
| 83.5 | 0.17584 | 0.19921 |
| 84.0 | 0.17341 | 0.19770 |
| 84.5 | 0.17140 | 0.19617 |
| 85.0 | 0.16982 | 0.19457 |
| 85.5 | 0.16864 | 0.19288 |
| 86.0 | 0.16780 | 0.19107 |
| 86.5 | 0.16721 | 0.18912 |
| 87.0 | 0.16674 | 0.18704 |
| 87.5 | 0.16630 | 0.18485 |
| 88.0 | 0.16580 | 0.18258 |
| 88.5 | 0.16520 | 0.18025 |
| 89.0 | 0.16449 | 0.17792 |
| 89.5 | 0.16370 | 0.17562 |
| 90.0 | 0.16284 | 0.17338 |
| 90.5 | 0.16193 | 0.17126 |
| 91.0 | 0.16095 | 0.16927 |
| 91.5 | 0.15987 | 0.16744 |
| 92.0 | 0.15867 | 0.16578 |
| 92.5 | 0.15733 | 0.16426 |
| 93.0 | 0.15584 | 0.16284 |
| 93.5 | 0.15425 | 0.16147 |
| 94.0 | 0.15259 | 0.16008 |
| 94.5 | 0.15093 | 0.15865 |
| 95.0 | 0.14932 | 0.15718 |
| 95.5 | 0.14776 | 0.15570 |
| 96.0 | 0.14625 | 0.15424 |
| 96.5 | 0.14479 | 0.15274 |
| 97.0 | 0.14333 | 0.15114 |
| 97.5 | 0.14187 | 0.14942 |
| 98.0 | 0.14037 | 0.14759 |
| 98.5 | 0.13885 | 0.14569 |
| 99.0 | 0.13728 | 0.14377 |
| 99.5 | 0.13569 | 0.14192 |
| 100.0 | 0.13406 | 0.14023 |
| 100.5 | 0.13241 | 0.13875 |
| 101.0 | 0.13074 | 0.13753 |
| 101.5 | 0.12906 | 0.13657 |
| 102.0 | 0.12739 | 0.13581 |
| 102.5 | 0.12572 | 0.13520 |
| 103.0 | 0.12406 | 0.13466 |
| 103.5 | 0.12242 | 0.13413 |
| 104.0 | 0.12080 | 0.13357 |
| 104.5 | 0.11921 | 0.13295 |
| 105.0 | 0.11765 | 0.13226 |
| 105.5 | 0.11612 | 0.13151 |
| 106.0 | 0.11462 | 0.13069 |
| 106.5 | 0.11314 | 0.12984 |
| 107.0 | 0.11168 | 0.12896 |
| 107.5 | 0.11025 | 0.12808 |
| 108.0 | 0.10887 | 0.12722 |
| 108.5 | 0.10752 | 0.12638 |
| 109.0 | 0.10620 | 0.12557 |
| 109.5 | 0.10493 | 0.12474 |
| 110.0 | 0.10369 | 0.12384 |
| 110.5 | 0.10249 | 0.12283 |
| 111.0 | 0.10133 | 0.12176 |
| 111.5 | 0.10019 | 0.12069 |
| 112.0 | 0.09909 | 0.11967 |
| 112.5 | 0.09802 | 0.11872 |
| 113.0 | 0.09698 | 0.11784 |
| 113.5 | 0.09596 | 0.11703 |
| 114.0 | 0.09497 | 0.11629 |
| 114.5 | 0.09401 | 0.11561 |
| 115.0 | 0.09307 | 0.11500 |
| 115.5 | 0.09216 | 0.11444 |
| 116.0 | 0.09126 | 0.11393 |
| 116.5 | 0.09039 | 0.11348 |
| 117.0 | 0.08954 | 0.11308 |
| 117.5 | 0.08870 | 0.11272 |
| 118.0 | 0.08789 | 0.11240 |
| 118.5 | 0.08709 | 0.11211 |
| 119.0 | 0.08631 | 0.11182 |
| 119.5 | 0.08554 | 0.11152 |
| 120.0 | 0.08478 | 0.11118 |
| 120.5 | 0.08404 | 0.11078 |
| 121.0 | 0.08332 | 0.11031 |
| 121.5 | 0.08260 | 0.10976 |
| 122.0 | 0.08190 | 0.10915 |
| 122.5 | 0.08121 | 0.10851 |
| 123.0 | 0.08052 | 0.10786 |
| 123.5 | 0.07985 | 0.10724 |
| 124.0 | 0.07919 | 0.10667 |
| 124.5 | 0.07853 | 0.10612 |
| 125.0 | 0.07789 | 0.10552 |
| 125.5 | 0.07725 | 0.10479 |
| 126.0 | 0.07662 | 0.10394 |
| 126.5 | 0.07600 | 0.10303 |
| 127.0 | 0.07538 | 0.10216 |
| 127.5 | 0.07478 | 0.10136 |
| 128.0 | 0.07417 | 0.10064 |
| 128.5 | 0.07358 | 0.09998 |
| 129.0 | 0.07299 | 0.09939 |
| 129.5 | 0.07241 | 0.09884 |
| 130.0 | 0.07183 | 0.09834 |
| 130.5 | 0.07127 | 0.09786 |
| 131.0 | 0.07070 | 0.09742 |
| 131.5 | 0.07014 | 0.09699 |
| 132.0 | 0.06959 | 0.09659 |
| 132.5 | 0.06904 | 0.09620 |
| 133.0 | 0.06850 | 0.09582 |
| 133.5 | 0.06797 | 0.09544 |
| 134.0 | 0.06744 | 0.09507 |
| 134.5 | 0.06691 | 0.09468 |
| 135.0 | 0.06639 | 0.09429 |
| 135.5 | 0.06588 | 0.09389 |
| 136.0 | 0.06537 | 0.09347 |
| 136.5 | 0.06487 | 0.09304 |
| 137.0 | 0.06437 | 0.09260 |
| 137.5 | 0.06388 | 0.09214 |
| 138.0 | 0.06339 | 0.09166 |
| 138.5 | 0.06291 | 0.09117 |
| 139.0 | 0.06243 | 0.09067 |
| 139.5 | 0.06196 | 0.09016 |
| 140.0 | 0.06149 | 0.08964 |
| 140.5 | 0.06102 | 0.08911 |
| 141.0 | 0.06057 | 0.08857 |
| 141.5 | 0.06011 | 0.08803 |
| 142.0 | 0.05966 | 0.08748 |
| 142.5 | 0.05922 | 0.08694 |
| 143.0 | 0.05878 | 0.08639 |
| 143.5 | 0.05834 | 0.08584 |
| 144.0 | 0.05791 | 0.08529 |
| 144.5 | 0.05749 | 0.08475 |
| 145.0 | 0.05706 | 0.08421 |
| 145.5 | 0.05664 | 0.08368 |
| 146.0 | 0.05623 | 0.08315 |
| 146.5 | 0.05582 | 0.08262 |
| 147.0 | 0.05542 | 0.08211 |
| 147.5 | 0.05502 | 0.08160 |
| 148.0 | 0.05462 | 0.08109 |
| 148.5 | 0.05423 | 0.08059 |
| 149.0 | 0.05384 | 0.08010 |
| 149.5 | 0.05345 | 0.07961 |
| 150.0 | 0.05307 | 0.07913 |
| 150.5 | 0.05269 | 0.07865 |
| 151.0 | 0.05232 | 0.07818 |
| 151.5 | 0.05195 | 0.07771 |
| 152.0 | 0.05158 | 0.07725 |
| 152.5 | 0.05122 | 0.07679 |
| 153.0 | 0.05086 | 0.07633 |
| 153.5 | 0.05050 | 0.07588 |
| 154.0 | 0.05015 | 0.07543 |
| 154.5 | 0.04980 | 0.07499 |
| 155.0 | 0.04946 | 0.07455 |
| 155.5 | 0.04911 | 0.07411 |
| 156.0 | 0.04878 | 0.07368 |
| 156.5 | 0.04844 | 0.07325 |
| 157.0 | 0.04811 | 0.07282 |
| 157.5 | 0.04778 | 0.07240 |
| 158.0 | 0.04745 | 0.07198 |
| 158.5 | 0.04713 | 0.07157 |
| 159.0 | 0.04681 | 0.07116 |
| 159.5 | 0.04649 | 0.07076 |
| 160.0 | 0.04618 | 0.07036 |
| 160.5 | 0.04586 | 0.06998 |
| 161.0 | 0.04556 | 0.06960 |
| 161.5 | 0.04525 | 0.06923 |
| 162.0 | 0.04495 | 0.06887 |
| 162.5 | 0.04465 | 0.06852 |
| 163.0 | 0.04435 | 0.06818 |
| 163.5 | 0.04406 | 0.06785 |
| 164.0 | 0.04376 | 0.06753 |
| 164.5 | 0.04348 | 0.06723 |
| 165.0 | 0.04319 | 0.06693 |
| 165.5 | 0.04290 | 0.06665 |
| 166.0 | 0.04262 | 0.06638 |
| 166.5 | 0.04234 | 0.06612 |
| 167.0 | 0.04207 | 0.06587 |
| 167.5 | 0.04179 | 0.06563 |
| 168.0 | 0.04152 | 0.06540 |
| 168.5 | 0.04125 | 0.06518 |
| 169.0 | 0.04099 | 0.06496 |
| 169.5 | 0.04072 | 0.06475 |
| 170.0 | 0.04046 | 0.06454 |
| 170.5 | 0.04020 | 0.06434 |
| 171.0 | 0.03994 | 0.06414 |
| 171.5 | 0.03969 | 0.06395 |
| 172.0 | 0.03943 | 0.06375 |
| 172.5 | 0.03918 | 0.06356 |
| 173.0 | 0.03893 | 0.06337 |
| 173.5 | 0.03869 | 0.06317 |
| 174.0 | 0.03844 | 0.06298 |
| 174.5 | 0.03820 | 0.06278 |
| 175.0 | 0.03796 | 0.06258 |
| 175.5 | 0.03772 | 0.06238 |
| 176.0 | 0.03748 | 0.06218 |
| 176.5 | 0.03725 | 0.06197 |
| 177.0 | 0.03702 | 0.06176 |
| 177.5 | 0.03679 | 0.06154 |
| 178.0 | 0.03656 | 0.06133 |
| 178.5 | 0.03633 | 0.06110 |
| 179.0 | 0.03611 | 0.06088 |
| 179.5 | 0.03588 | 0.06065 |
| 180.0 | 0.03566 | 0.06041 |
